# Effect of Ti and Au buffer layers on controlling the density and wettability of well-aligned ZnO nanorod arrays grown on different substrates†

**DOI:** 10.1039/d3na00299c

**Published:** 2023-06-28

**Authors:** M. Kamruzzaman, J. A. Zapien

**Affiliations:** a Department of Physics Begum Rokeya University Rangpur Rangpur-5400 Bangladesh kzaman.phy11@gmail.com +880-1771034439; b Department of Materials Science and Engineering, Center Of Super-Diamond and Advanced Films (COSDAF), City University of Hong Kong Hong Kong SAR P. R. China apjazs@cityu.edu.hk +852-3442-7823

## Abstract

ZnO nanorod arrays (NRAs) have potential applications as building blocks for nanoscale electronic, optoelectronic, and sensing applications. The density of ZnO NRAs is controlled by a simple low-cost hydrothermal growth process. It is shown that Ti and Au thin buffer layers can be used to control ZnO NRA density up to an order of magnitude on a wide variety of substrates including bare glass AZO, ZnO seeded AZO, FTO and ITO substrates, respectively. We investigate surface morphological, structural and optical properties of ZnO NRAs by field emission scanning electron microscopy, transmission electron microscopy, X-ray diffraction, Raman, and photoluminescence spectroscopy measurements, respectively. To highlight the importance of NRA density, wettability measurements show large dependence on density and static water contact angles range from as low as ∼23° to as large as ∼142°. These results indicate that the capability to control the density of ZnO NRAs, and thus their wettability, can have additional implications such as in their use in biosensors, field emission, dye-sensitized solar-cells (DSSCs), and photocatalytic activity in addition to potential light trapping effects over wide spectral ranges.

## Abstract 2

ZnO nanorod arrays (NRAs) have potential applications as building blocks for nanoscale electronic, optoelectronic, and sensing applications.

## Graphical abstract

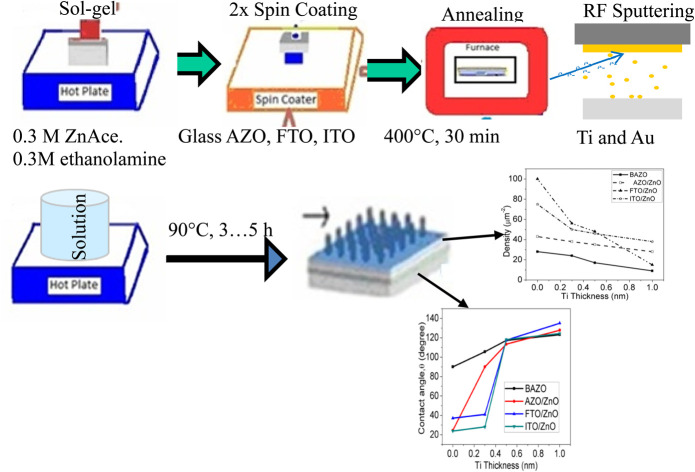

## 1. Introduction

ZnO NRAs show great potential applications for next generation nanodevices.^1–4^ Among CdO, WO_3_, TiO_2_, SnO_2,_ MgO, In_2_O_3_ and ZnO metal oxides,^5^ ZnO is widely used. ZnO nanostructures are manipulated as key materials in solar cells,^6,7^ electromechanical devices,^8^ ultraviolet (UV) lasers,^9^ light-emitting and Schottky diodes,^10^ field emission devices,^11^ high performance nanosensors,^12^ piezoelectric nanogenerators,^13^ biosensors,^14^ nanopiezotronics,^15^ surface acoustic wave devices,^16^ flat panel displays,^17^ quantum dot devices,^18^ bio safety and bio-compatibility^19^ due to their intrinsic properties of non-toxicity, and good electrical and optical features. To date, intensive research has been focused on the fabrication of ZnO nanostructures and in correlating the resulting morphology with their size-related mechanical, chemical, optical, and electrical properties.^20^ Although various kinds of ZnO nanostructures can be realized, ZnO nanorods (NRs) have been extensively studied because of their easy fabrication and relatively simple device applications^21^ as interconnects and functional units.^9,22^ For proper device construction and application a precise control of ZnO NRA density on different substrates is very important because they are directly related to how ZnO NRAs interact with each other to control the aforementioned properties for such device applications.^21^ The density control of ZnO NRAs in a specific position on the Si substrate offers a great functional component in Si- based optoelectronic devices.^23^ For example, high-density NRAs are more efficient for photocatalytic devices rather than low-density ones. However, low-density NRAs are better for field emission devices than high-density NRAs because the density of highly densely packed NRAs greatly reduces the field enhancement effect.^24^ It should be noted that ZnO NRA density control is very difficult without prepatterning seed or catalyst dots. To control the vertical alignment of ZnO NRA density, so far two strategies have mostly been implemented.^24^ The first strategy includes e-beam lithography,^25^ laser interferometry,^26^ and nanoimprinting.^27^ They produce high-quality NRs with well-ordered distribution notwithstanding complicated equipment, high reaction temperature, and rigorous conditions needed for their growth. On the other hand, the second strategy group produces a random arrangement of nanorod arrays^13,28,29^ which are inexpensive and easily scalable. There are a few reports of ZnO NRA density growth using a hydrothermal growth process, but they all have some limitations because the ZnO seed layer still serves to grow highly dense NRAs.^30–34^

Wettability is an important fundamental property of solid materials. Wettability control has received increasing interest for its many potential applications from self-cleaning coatings to protein absorption, drag reduction, cell adhesion, anti-corrosion, micro channels, waterproof devices and other non-wetting related applications.^35–39^ Wettability mainly depends on surface energy and roughness. The composition segregation, surface restructuring and structure relaxation can reduce the surface energy resulting in an increased contact angle.^40^ UV light irradiation or storage in the dark can also significantly increase the contact angle.^22^ It is well known that smooth low energy surfaces exhibit contact angles up to 120°,^41,42^ however natural lotus leaves demonstrate a water contact angle (WCA) of 160°.^43^ The most pronounced morphologies for superhydrophobic applications, ZnO nanorods/nanowires have been used to change the surface characteristics of textiles, polymers, dye synthesized solar cells and so forth.^43^ A recent report has demonstrated that ZnO NRAs can be used for waterproof, transparent, and flexible devices.^44^ The wettability of ZnO NRAs grown on Si, glass, AZO, and sapphire substrates has been reported and shown that the maximum contact angle lies in between 104 and 166°.^42,49–51^ Although the superhydrophobicity nature has been described in terms of roughening;^45–48^ the effect of the synthesis parameters on the hydrophobicity is still a serious challenge.^45–48^ In the literature, no such reports are available regarding wettability change with ZnO NRA density variation on different substrates grown by the hydrothermal growth process.

An effective growth approach and a facile control of distribution, alignment and density of ZnO NRAs are prerequisites for their applications.^21^ Over the past few years, various methods have been used to synthesize one dimensional (1D) ZnO nanostructures such as thermal decomposition of precursors,^52^ different chemical vapor deposition processes,^53^ pulsed laser deposition,^54^ molecular beam epitaxy,^49^ vapor phase transport technique,^55^ magnetron sputtering,^56^ oxidation of zinc metal^57^ and metal–organic vapor phase.^58^ These methods are expensive, require moderate to high temperature, stringent experimental conditions and sometimes dangerous chemicals are also used to grow NRAs on a commercial scale.^59^ On the other hand, ZnO NRA growth based on the hydrolysis process is simple, cheaper and does not require stringent experimental conditions.^60^ This technique can be used for the growth of well-oriented 1D ZnO NRs on a large range of substrates and, importantly, enables the possibility to overcome the solubility limit by using different types of solvents (water, ethanol, methanol, *etc.*), reduction reagents (NH_4_OH, NaOH, KOH and KCl *etc.*) as well as easily hydrolysable amines (urea, hexamethylenetetramine),^61,62^ and the impurity concentration can be controlled precisely.^63^ In addition, this technique can be used for large-scale area deposition in, for example, low cost solar cell mass production.

In this paper, we present a systematic study on the ability to control the density of ZnO NRAs using Ti and Au buffer layers on different seeded and unseeded substrates *via* a facile low temperature, <100 °C, hydrothermal growth process.

## 2. Experimental details

### 2.1 Materials and seed layer solution

To synthesize ZnO NRAs, a two-step hydrolysis process was employed. In the first step, a seed layer sol gel solution was prepared by dissolving an equimolar concentration of zinc acetate dihydrate (Zn(CH_3_COO)_2_·2H_2_O), 0.3 M and monoethanolamine (MEA), 0.3 M in ethanol. The solution was heated at 70 °C with constant vigorous stirring for 1 h, then the solution was stirred overnight at room temperature (RT). The solution was then spun onto glass AZO (aluminium doped zinc oxide), FTO (fluorine doped tin oxide), and ITO (indium doped tin oxide) substrates at 2.5k rpm for 60 s and annealed at 250° for 10 min in the air. The process was repeated twice to make a uniform and complete film. After successive coating the films were annealed at 400 °C for 30 min to remove residual organic solvents and form ZnO crystalline seed layers. The buffer layers of Ti (0.0, 0.3, 0.5 and 1.0 nm) and Au (4.0, 8.0, 12.0 and 16.0 nm) were deposited on bare AZO, ZnO seeded AZO, FTO, and ITO substrates by a magnetron sputtering system with a pressure of 3 × 10^−7^ Torr, DC voltage 270 and 300 V, respectively.

All chemicals used in this work were purchased from Sigma-Aldrich, USA based on the highest available purity. Prior to the deposition of seed layers the substrates were subsequently cleaned in Decon 90, acetone, ethanol and deionized (DI) water with the assistance of ultrasonic agitation each for 15 minutes and dried in nitrogen gas flow, and finally cleaned in a UV-Ozone cleaner for 10 min.

### 2.2 ZnO NRA growth conditions

ZnO NRAs was grown on the Ti and Au buffer layers deposited bare AZO, AZO/ZnO, FTO/ZnO and ITO/ZnO substrates by suspending in a solution prepared from an equimolar concentration of zinc nitrate hexahydrate and hexamethylenetetramine. Zinc nitrate hexahydrate (25 mM) and hexamethylenetetramine (25 mM) were dissolved in 200 ml deionized water, the substrates were made to float face down in a quartz beaker with the help of kapton tape and the beaker was covered with aluminum foil. After that, the beaker was directly inserted in a preheated water containing beaker at 90 °C for 5 hours to induce the growth of nanorods. After the growth induction time, the beaker was taken out and the substrates were washed several times with deionized water to remove any residual salts and were dried in nitrogen flow gently.

### 2.3 Characterization

The surface morphology and the cross-section of ZnO NRAs were characterized by field emission scanning electron microscopy (FE-SEM) (Philips XL30 FEG). Transmission electron microscopy (TEM) images were collected using a high resolution TEM instrument with 200 keV (Philips FEG TEM CM200). X-ray diffraction (analytical Philips X'Pert diffractometer) data were taken in the range of 20° to 70° with a step width of 0.02°. Raman and photoluminescence (PL) spectra were recorded using a Renishaw-Invia micro-Raman spectroscope (excitation wavelength 514 nm) and Renishaw-Invia UV/Vis multiple laser Raman (excitation wavelength 244 nm) spectroscopies, respectively. The static water contact angle (WCA) measurements were made by dropping ∼ 4 μL of deionized (DI) water on ZnO NR arrays and the images were collected with a digital camera system (Canon 5D and Canon EF 100 mm f/2.8 Macro USM Lens), and the values of the static water contact angle were determined using CorelDRAW angular dimension software by considering the force balance at the three-phase contact line where the droplet edge was bent. The peak position, full width at half maximum (FWHM) and the integrated intensity of the XRD, Raman and PL spectra were found out by the Lorentzian fitting with the experimental data.

## 3. Results and discussion

### 3.1 Surface morphology by SEM

**Fig. 1:**
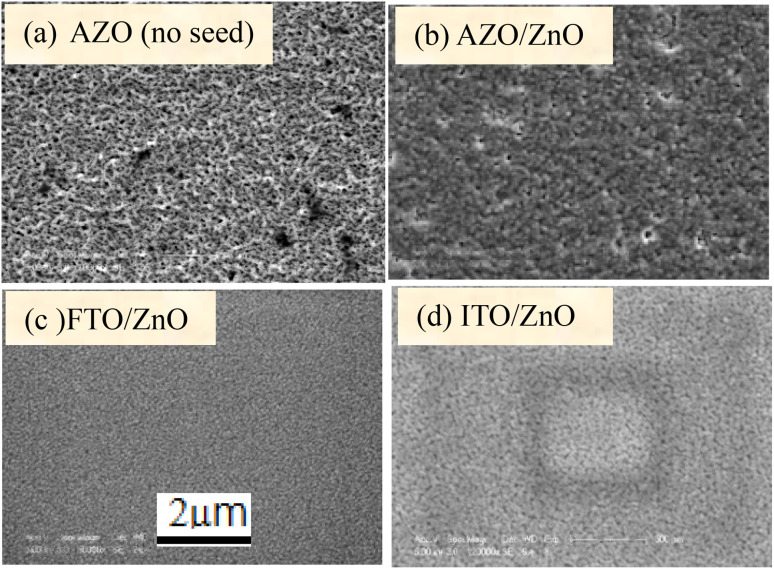
Surface morphology of (a) bare AZO, (b) AZO/ZnO, (c) FTO/ZnO and (d) ITO/ZnO substrates (scale bar 2 μm).

Fig. 1(a)–(d) show the surface morphology of bare AZO, and ZnO coated AZO/ZnO, FTO/ZnO and ITO/ZnO, respectively. The surface morphology of bare AZO is more rough with non-uniform large grain size distributed over the whole substrate surface rather than the surface morphology of AZO/ZnO.

**Fig. 2:**
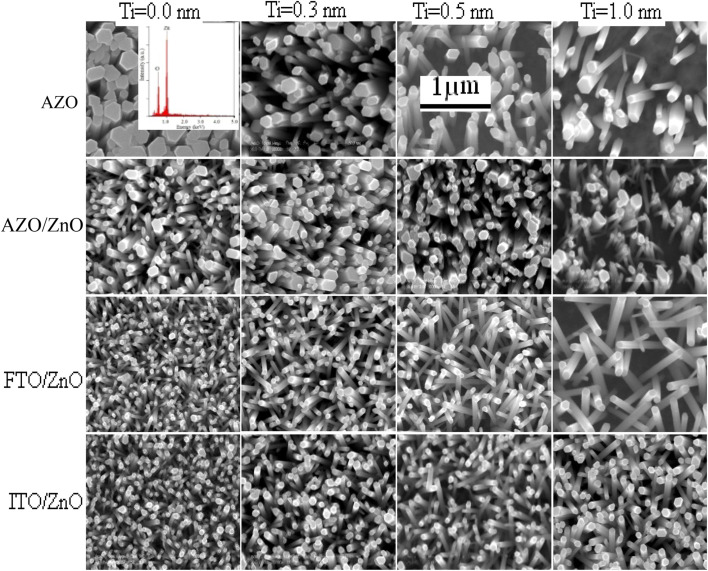
Top-view FE-SEM images of ZnO NRAs on bare AZO (1^st^ row), AZO/ZnO (2^nd^ row), FTO/ZnO (3^rd^ row), and ITO/ZnO (4^th^ row) for the Ti buffer layer of different thicknesses. No Ti buffer, 0.0 nm Ti, in the left-most column and increasing Ti buffer layer thickness of columns 2 to 4 corresponding to the Ti thickness of 0.3, 0.5, and 1.0 nm, respectively (scale bar 1 μm).

**Fig. 3:**
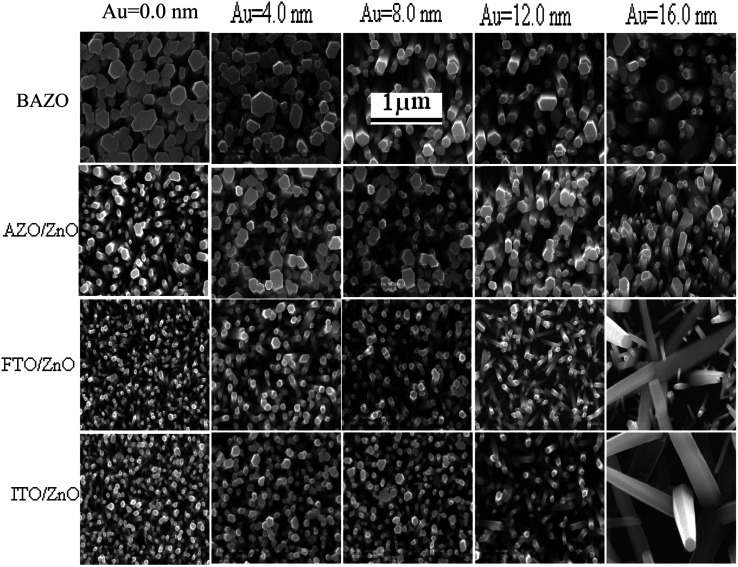
Top view FE-SEM images of ZnO NRAs on bare AZO (1^st^ row), AZO/ZnO (2^nd^ row), FTO/ZnO (3^rd^ row), and ITO/ZnO (4^th^ row) for the Au buffer layer of different thicknesses. No-Au buffer, 0.0 nm Au, in the left-most column and increasing Au buffer layer thickness in the case of columns 2 to 5 corresponding to the Au thickness of 4.0, 8.0, 12.0, and 16.0 nm, respectively (scale bar 1 μm).

FTO/ZnO and ITO/ZnO substrates seem to be smoother surfaces with uniform smaller grain size distributed on the surfaces. The effect of the seed layer nature on the ZnO nanorod array growth has been described in our previous article.^30^ The inhibitor buffer layers of Ti (0.0, 0.3, 0.5, and 1.0) nm and Au (4.0, 8.0, 12.0, and 16.0) nm were deposited on bare AZO, AZO/ZnO, FTO/ZnO and ITO/ZnO surfaces which are the control data, and ZnO NR arrays were grown on these substrates as shown in Fig. 2 and 3, respectively.

**Fig. 4:**
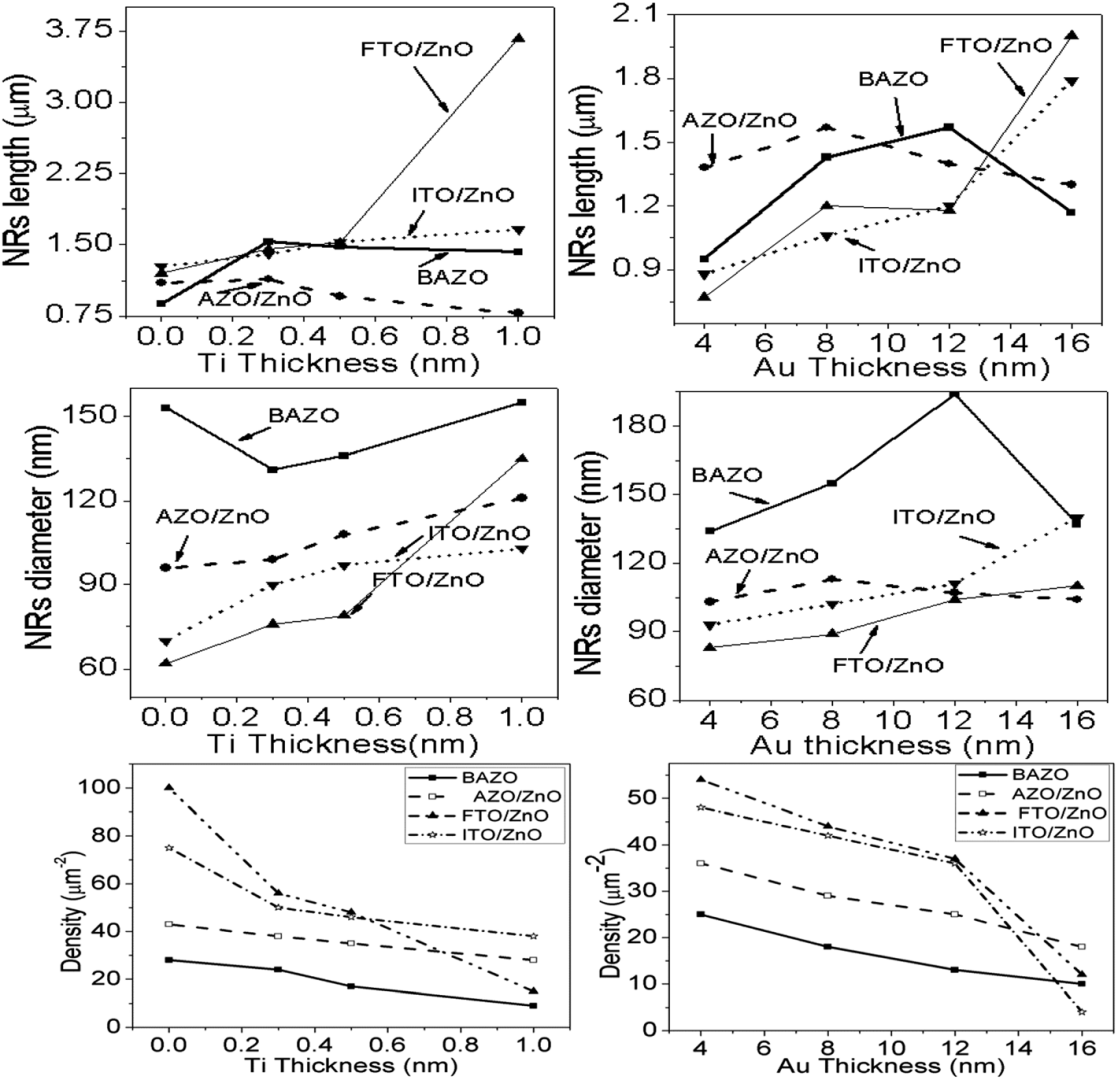
Variation of length, diameter and density of ZnO NRAs with the variation of buffer layer thickness of (i) Ti, left column and (ii) Au, right column, respectively.

Each row depicts the results for one of the four different substrates used, namely bare AZO (1^st^ row in Fig. 2 and 3), and substrates seeded with ZnO nanocrystals presented in the 2^nd^ to 4^th^ rows, respectively. The left column corresponds to Ti = 0.0 nm (no-Ti buffer, or 0.0 nm Ti) and increasing Ti buffer layer thickness for columns 2 to 4 corresponding to Ti thickness of 0.3, 0.5 and 1.0 nm. Similarly, for Au buffer layers the columns 1 to 5 correspond to an Au thickness of 0.0, 4.0, 8.0, 12.0 and 16.0 nm (Fig. 3). The corresponding cross-section of the grown ZnO NR arrays on these substrates is presented in ESI Fig. S1 and S2† which also include (inset) the digital images of water droplets used for the contact angle measurements. The average areal number of NR array density was calculated at high magnification (80 000×, not shown here) by counting the number of NR tips that appeared on the top surface at five substrate locations (μm^−2^) chosen randomly^28^ and the obtained density is given in Tables S1 and S2.† It is clearly seen from Fig. 2, 3, and Tables S1 and S2† that the density of ZnO NRAs changes considerably with an increase of Ti and Au thicknesses. However, the density of ZnO NRAs grown on the bare AZO substrate (1^st^ row of Fig. 2 and 3) is lower than that grown on the AZO/ZnO seeded substrate (2^nd^ row of Fig. 2 and 3) while their average diameter is larger than the NRs grown on AZO/ZnO. It is expected that the AZO substrate surface acts as a self-seeded layer with larger ZnO grain size compared to AZO/ZnO. The density of ZnO NRAs noticeably changes from ∼28 to 9 μm^−2^ and 25 to 10 μm^−2^ for bare AZO and from 43 to 28 μm^−2^ and 36 to 18 μm^−2^ for AZO/ZnO (1^st^ and 2^nd^ rows of Fig. 2 and 3, Tables S1 and S2†) with the increase of Ti and Au thickness. It should be noted that the length of NRs slightly decreases with an increase of Au film thickness from 12.0 to 16.0 nm attributed to an increase in lattice mismatch with the substrate, which verifies the strong influence of AZO surface morphology.^64,65^ The obtained results (Tables S1 and S2†) are in good agreement with the reports,^66–70^ where ZnO NRAs were grown on AZO/Au. The length of NRs increases up to a thickness of 8.0 nm for Au and 0.5 nm for Ti in the case of AZO and AZO/ZnO, after which the length decreases due to increase in buffer layer thickness (∼750 nm of AZO), which is a limiting factor of AZO and AZO/ZnO (Fig. 4). On the other hand, the density of NRAs grown on FTO/ZnO changed from ∼100 to 15 μm^−2^ for Ti and 54 to 12 μm^−2^ for Au while it decreased from ∼75 to 38 μm^−2^ and 48 to 4 μm^−2^ for ITO/ZnO (Tables S1 and S2†), respectively. From these results it is evident that NRA density grown on ZnO coated FTO and ITO substrates is ∼10 times lower than the initial NRA density and with more uniform distribution. When the seed layers were coated with Au and Ti buffer layers the density of ZnO NRAs decreased with an increase of Ti and Au thickness, which might inhibit the growth of ZnO NRs.^69,70^ According to ref. 68, the lateral growth is not as effectively suppressed at lower nucleation density, resulting in larger diameter NRs.

We have reduced the number of nucleation sites of ZnO seeds by varying the thickness of Ti and Au. As the thickness is increased the nucleation site of ZnO seeds possibly decreased and consequently growth in NRA density decreased, and hence the diameter of ZnO NRs changed accordingly (Fig. 4). It is also seen that the tips of several NRs touched and/or crossed each other when the density (samples: Au (12.0 nm)/ZnO/FTO, Au (16.0 nm)/ZnO/ITO) is much lower, which could be due to the piezoelectric properties of ZnO NRs and/or the slightly tilted growth at the initial stage, which lead to an increase in the diameter of the NRs.^70^ In the EDX spectrum (inset Fig. 2), only Zn and O atoms are detected. No evidence of other impurities was found in the EDX spectrum, demonstrating that the grown NRs are of pure ZnO. From the above discussion, it is clear that the density of ZnO NRAs could effectively be controlled using Ti or Au buffer layers. Although the density of ZnO NR arrays could be controlled, simultaneous control of nanostructure morphology, andaspect ratio (length/diameter) are challenging issues to achieve using the facile hydrothermal method.^28,34,66,67^

### 3.2 Transmission electron microscopy (TEM)

The crystallinity, defects and atomic structure of the individual ZnO nanorods were investigated using high resolution TEM and selected area electron diffraction (SAED) measurements.

**Fig. 5:**
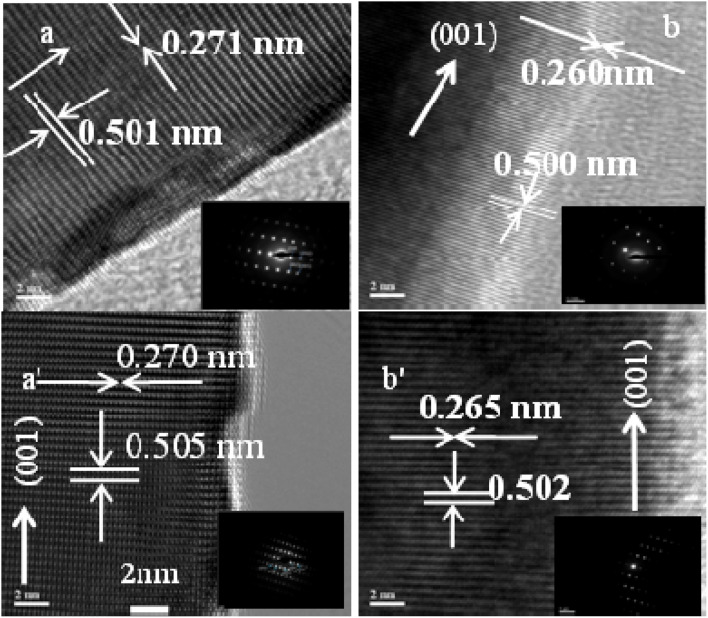
TEM images top row for (a) Ti = 0.0, (b) Ti = 1.0 nm and bottom row for (a′) Au = 0.0, (b′) Au = 16.0 nm (scale bar 2 nm).

Fig. 5 shows high resolution TEM (HRTEM) and selected area electron diffraction (SAED, inset) of ZnO NRs for Ti (0.0 and 0.5 nm, top row) and Au (0.0 and 12.0 nm, bottom row), respectively. From the inset figures (top and bottom rows) single crystalline properties of non-defect sites with *c*-axis orientation can be seen. The HRTEM observations of Fig. 5 display the average lattice constant ∼0.502 nm corresponding to the (002) plane and the fringes are separated by an average of 0.265 nm indicating *d*-spacing, respectively, which confirmed that the grown ZnO NRs are preferentially oriented along the *c*-axis, *i.e.*, the [0001] growth direction. No change is observed in the growth direction due to Ti and Au films. The presence of the straight line diffraction lattice points in SAED patterns indicated that the grown NRs have a single crystalline growth along the direction [0001] (Fig. 5, insets).

### 3.3 X-ray diffraction

**Fig. 6:**
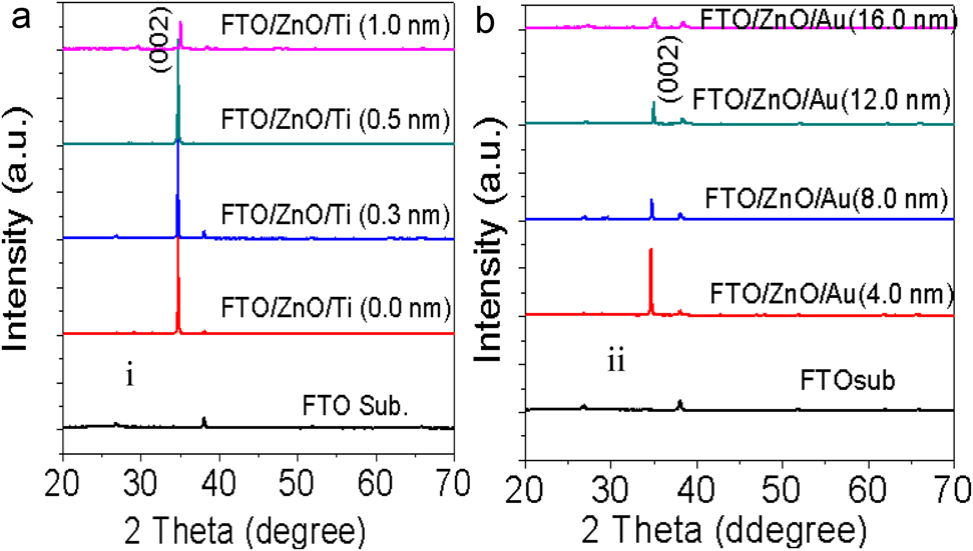
XRD patterns of ZnO NRAs grown on (a) FTO/ZnO/Ti and (b) FTO/ZnO/Au buffer layer.

To check the crystallinity status of the ZnO NRAs, the XRD data were collected for ZnO NRAs grown on (a) FTO/ZnO/Ti and (b) FTO/ZnO/Au (one series for Ti and Au buffer layers) substrates, shown in Fig. 6. A high intense sharp peak, (002), appears in each spectrogram. According to JCPDS card no. 036-1451, this diffraction peak is indexed for the hexagonal structure of ZnO attributed to the preferential vertical orientation along the (002) plane and [0001] direction normal to the substrate surface.^71,72^ It can be seen that the overall intensity of the (002) peak decreases with the increase of Ti and Au thickness, which could be due to the decrease of NR size and/or NRA density. However, no extra peak is observed suggesting that Ti and Au were not incorporated into the wurtzite ZnO structure.^68–70^ But we are not sure whether at least a part of Ti or Au was incorporated into the ZnO crystal because the X-ray diffractometer cannot detect a component in a mixture for which the whose quantity is approximately less than 1%. The peak position of the (002) peak is slightly shifted to a higher value, which could be attributed to the increase of NR diameter or grain size (Tables 1 and 2) with the increase of Ti and Au buffer thickness. The lattice constants (*a*, *c*) for the (100) and (002) adjacent planes, and the average grain size (*L*_g_) were calculated using relationships (1) and Scherrer formula (2), respectively,1
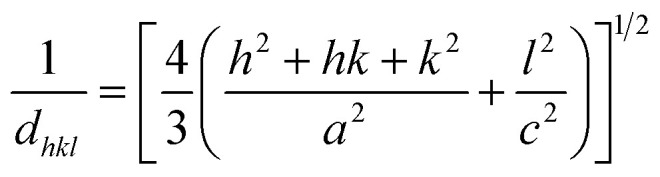
and,2
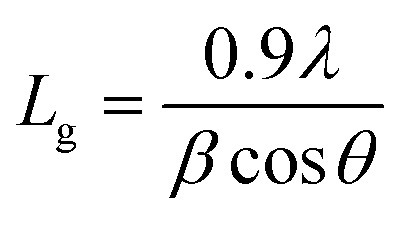


**Table 1:** Lattice constants, grain size, and *c*/*a* of ZnO NRs for Ti buffer layers

Substrate	Ti thickness (nm)	Lattice constant (Å)		Grain size (nm)	*c*/*a*
		*a*	*c*		
AZO		3.420	5.208		
FTO		10.248	—		
ITO		4.748	3.200		
Bare AZO	0.0	3.303	5.163	65.5	1.563
	1.0	3.232	5.187	78.3	1.605
AZO/ZnO	0.0	3.239	5.082	40.5	1.569
	1.0	3.289	5.152	118	1.566
FTO/ZnO	0.0	3.294	5.158	86.6	1.565
	1.0	3.259	5.147	119	1.570
ITO/ZnO	0.0	3.302	5.129	76	1.565
	1.0	3.296	5.162	78	1.566

**Table 2:** Lattice constants, grain size, and c/*a* of ZnO NRs for Au buffer layers

Substrate	Au thickness (nm)	Lattice constant (Å)		Grain size (nm)	*c*/*a*
		*a*	*c*		
Bare AZO	4	3.310	5.178	68.18	1.564
	16	3.287	5.148	78.4	1.566
AZO/ZnO	4	3.254	5.108	77.9	1.569
	16	3.285	5.176	84.5	1.562
FTO/ZnO	4	3.294	5.17	68.9	1.564
	16	3.284	5.105	74.84	1.603
ITO/ZnO	4	3.235	5.182	78.1	1.601
	16	3.333	5.139	87.3	1.538

The obtained values are given in Tables 1 and 2. It is seen that the lattice parameter ‘*c*’ determined from XRD data for the (002) plane is 0.520 nm which is in good agreement with the standard lattice constant and published values^69,73^ but there is no remarkable change in lattice constants due to the increase of Ti and Au buffer layer thickness. However, the lattice parameter (*c* = 0.514 nm) obtained from TEM-SAED images is comparable to value obtained from the XRD data. This discrepancy may be attributed to the diffraction phenomenon.^74^

Fig. S3† shows the position (1^st^ column), full width at half maximum (FWHM) (2^nd^ column) and the integrated intensity (3^rd^ column) of the (002) plane of NRs grown on the FTO/ZnO substrate for Ti (0.0 to 1.0 nm) and Au (4.0 to 16.0 nm), respectively. It is seen that the average value of FWHM slightly increases and the integrated intensity decreases with the increase of Ti and Au buffer layer thickness. The increase of FWHM value with the increase of Ti and Au buffer layer is attributed to the NR density or the size effect. Importantly, the low value of the FWHM (for Ti = 0.0 and Au = 0.0) indicates that the NRs grown without any buffer layer on FTO/ZnO have a better crystalline structure.

### 3.4 Raman spectroscopy

**Fig. 7:**
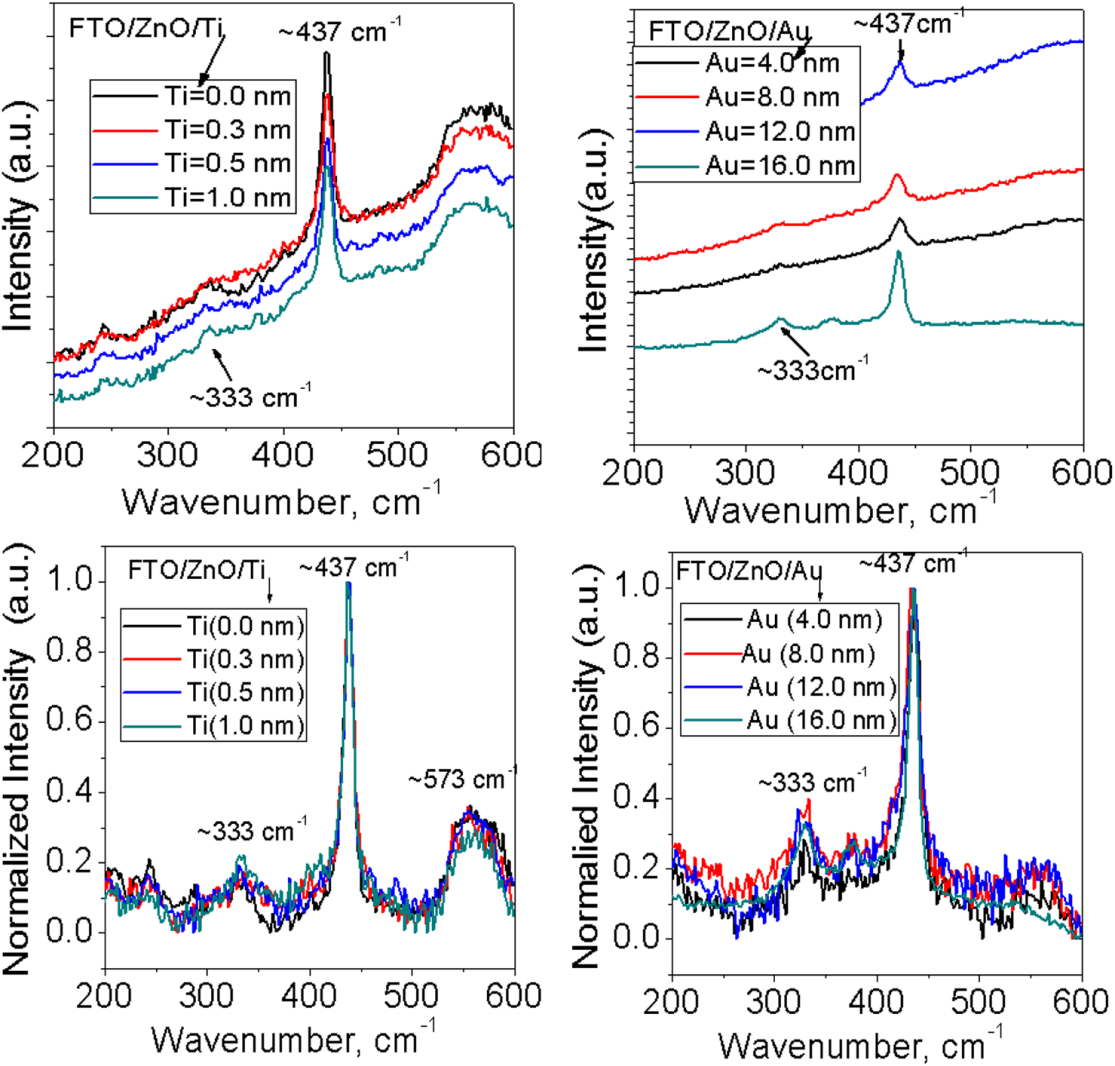
Raman spectra of ZnO NRAs grown on FTO/ZnO/Ti and FTO/ZnO/Au buffer layers: (i) top row normal and (ii) bottom row normalized intensity (after baseline subtraction).

Raman spectra of ZnO NRAs grown on (a) FTO/ZnO/Ti and (b) FTO/ZnO/Au are shown in Fig. 7 and S4.† A high intense peak is observed at 435–437 cm^−1^ in each spectrum which is attributed to a high frequency mode (E2 mode).^75^ This type of mode originates from the vibrations of oxygen atoms and confirm that the as-grown ZnO NRs are the wurtzite hexagonal phase with very good crystallinity.^75^ It is evident that the peak position E2 (high mode) (Fig. S4,† 1^st^ column) does not systematically shift with the increase of Ti and Au buffer layer thickness. However, the intensity decreases with an increase of Ti and Au thickness concomitant with the decrease of NRA density (Fig. 7). It is well known that the peaks position and profile of Raman peaks depend on several factors such as crystallization, structural disorder, crystal defects and residual stress in a sample as well as the density of ZnO NRs.^73^

To better clarify the results, the change of the full width at half maximum (FWHM) and the integrated intensity value of E2 peak were found out and the values of FWHM and the intensity do not follow any systematic rule with the increase of Ti and Au buffer layer thickness or the decrease of NRA density. But the low value of the FWHM of NRs grown on the FTO/ZnO substrate without Ti and Au buffer layer indicates that the NRs grown on FTO/ZnO have a better crystalline structure. Since the integrated intensity is highly dependent on the size of the ZnO NRs, the decrease of integrated intensity with the increase of Ti buffer layer is attributed to the NR arrays, *i.e.*, the NR size.

### 3.5 Photoluminescence (PL)

**Fig. 8:**
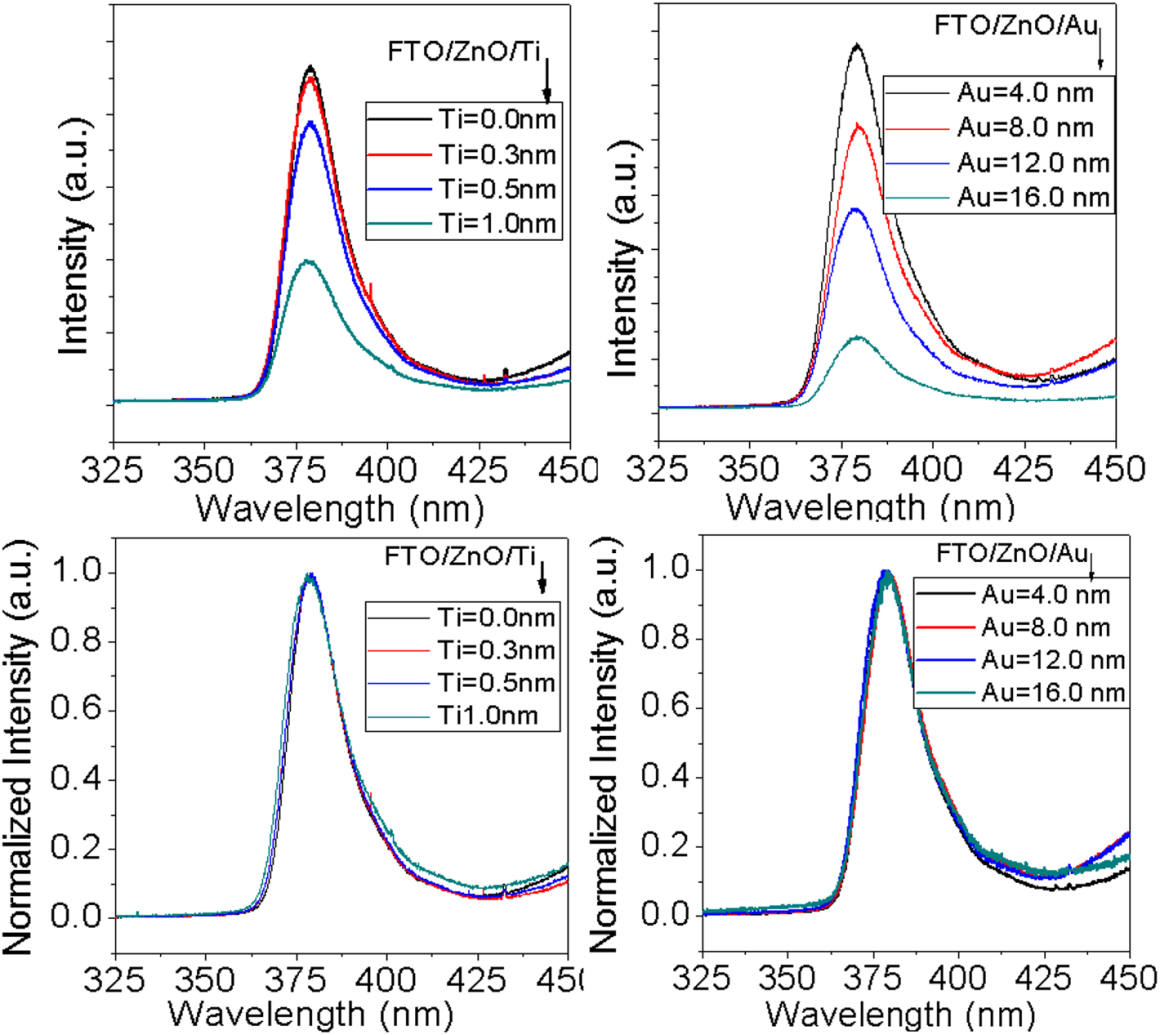
PL spectra of ZnO NRAs grown on (i) FTO/ZnO/Ti, and (ii) FTO/ZnO/Au buffer layer, (i) top row normal and (ii) bottom row normalized intensity (after baseline subtraction).

PL is an important characterization technique to investigate structural defects and the optical properties of nanostructure materials. It is well known that the UV emission in ZnO nanostructures arises from the recombination of free charge excitons^76^ and visible emissions attributed to any structural defects.^77^ In ZnO nanostructures the defects related luminescence may occur due to the oxygen vacancies which is explained by the radiative transitions between oxygen vacancies, interstitial zinc and deep acceptors (Zn vacancies).^78–80^Fig. 8 shows the PL spectra of ZnO NRAs grown on the FTO/ZnO substrate with the variation of Ti and Au thickness. A high-intensity UV emission peak is observed in each spectrum centered at ∼380 nm which is called near band edge (NBE) emission and the presence of this band is an indicator of the good crystallinity of ZnO NRs.^81^ The surface/interface states have an influence on the size of ZnO nanostructures^81^ and possibly contribute to the optical properties. The dimensions of the synthesized ZnO NRs (average diameter > 80.0 nm) are greater than the exciton Bohr radius 2.34 nm,^82^ so the size dependent quantum confinement effect has a low possibility of occurrence.^82^ From the normalized PL spectra of ZnO NR arrays (Fig. 8 bottom row) it can be seen that the NBE peak position of the samples doesn't significantly shift with the density and/or NR size variation. However, a non-systematic peak positon shifting may be attributed to the improper alignment of the laser excitation and the sample or long time switch on the laser source for many samples characterization. Fig. S5† shows FWHM (2^nd^ column) and integrated intensity (3^rd^ column) variation of NBE peak with Ti and Au thickness. Insignificant variation of FWHM and integrated intensity is observed. However, the lower value of FWHM and the higher PL intensity of ZnO NRs grown on unbuffered substrates are clear evidence of the better crystal quality of ZnO NRs.^83,84^

### 3.6 Static water contact angle

The water contact angle (WCA) of a smooth surface is limited to 120° (ref. 40) and further increasing the contact angle requires increasing the surface roughness and/or surface free energy.^85^ The ZnO NR arrays grow faster along the (002) plane due to their lower surface free energy^69,70^ and the roughness is associated with the ZnO NRA density. The variation of the water contact angle for a smooth surface can be described by Young's model as^86^

3 cos *θ*_*y*_ = (*γ*_sv_ − *γ*_sl_)/*γ*_lv_

where *γ*_lv_, *γ*_sl_ and *γ*_sv_ are the surface tension of liquid–vapour, solid–liquid, and solid–vapour, respectively. In fact, in nature a real perfectly flat where water can make 100% contact with a hydrophobic rough surface is difficult to find. Thus, eqn (3) proposed by Young does not consider the role of the surface roughness and hence the roughness ought to be considered while there must be bubbles trapped at the interface. The effect of a rough surface on wetting was included in the model proposed by Wenzel.^87^ According to this approach, a liquid drop completely fills the grooves of a rough surface and it is related as follows:^87^cos *θ*_rough_ = *r* cos *θ*_smooth_

4 cos *θ*_*y*_ = *r*(*γ*_sv_ − *γ*_sl_)/*γ*_lv_ = *r* cos *θ*_*y*_

where *r* is the surface roughness factor which is always greater than unity and depends on the change in surface roughness with hydrophobicity.^87^ This model assumes that the liquid can penetrate inside the rough surface and provides hydrophobic interfaces with contact angles less than 120°; however, it cannot impart superhydrophobicity,^88^ whereas Cassie–Baxter proposed a model where a liquid cannot penetrate inside a rough surface^89^according to the equation

5 cos *θ* = *f* (cos *θ*_*y* + 1_) − 1

where *f* is the area fraction of the actual solid surface area to the projected solid surface area. Therefore, the wettability of ZnO NRAs followed both Wenzel and Cassie–Baxter's models.^87,89^

**Fig. 9:**
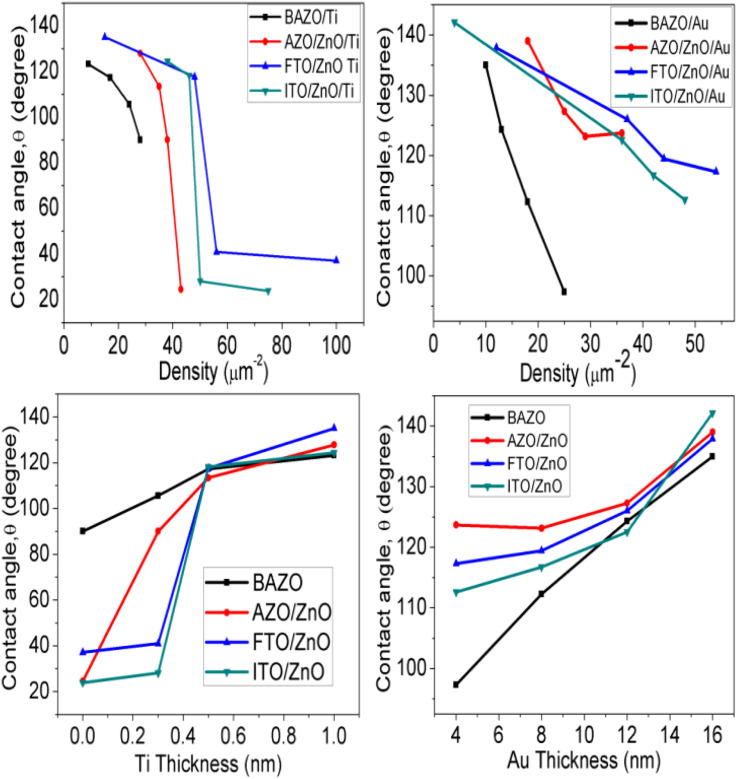
Variation of contact angle with ZnO NR array density (top row), Ti and Au buffer thickness (bottom row).

ESI Fig. S1 and S2† (inset) show the wettability measurement of ZnO NRAs under different substrate conditions. The change of the contact angle with NRA density and thickness variation is shown in Fig. 9. It is obvious that the contact angle changes from 90° to 123° (AZO), 24° to 127° (AZO/ZnO), 37° to 135° (FTO/ZnO), and 23° to 124° (ITO/ZnO) for the Ti buffer layer, while it changes from 97° to 135° (AZO), 123° to 139° (AZO/ZnO), 117° to 137° (FTO/ZnO), and 112° to 142° (ITO/ZnO) for the Au buffer layer, respectively.

It is seen that wettability changes from hydrophobicity to superhydrophobicity as the thickness of Ti and Au buffer layers increases resulting in a decrease in the density of ZnO NRAs. Low contact angles ≤90°are observed, as in the case of BAZO/Ti (0.0 nm) ∼90°, AZO/ZnO/Ti (0.0, 0.3 nm) ∼24°, 90°, FTO/ZnO/Ti (0.0, 0.3 nm) ∼37°, 40.0°, and ITO/ZnO/Ti (0.0, 0.3 nm) ∼23°, 28°, due to the high NRA density with small air grooves containing too limited air to support water droplets^49^ and hence the water droplet spreads on the samples, *i.e.*, water enters into the air grooves resulting in higher surface wettability due to the increase in contact area,^87^ in accordance with the Wenzel model contact angle limit (0–90°).^87^ As the NR array density decreases, the surface air grooves increase. These air grooves are filled with a large volume of air and the captive air in the grooves plays an important role in creating air/water interfaces, *i.e.*, to support the water droplet on it. So, it is understood that low density ZnO NR arrays with a large volume of captive air display less wettability properties.^49^ Thus, the obtained contact angle value in between 180 and 90° supports the Cassie–Baxter model.^89^ In this work ZnO NRAs show better hydrophobicity at lower density indicating that a lower density can provide a more appropriate proportion of air/water interfaces for maintaining water droplets on the ZnO NRA surface.^89,90^ From Fig. 9 it is clear that good hydrophobicity occurs at lower densities when Ti and Au buffer thickness is greater than 0.5 and 12.0 nm. The maximum water contact angle is obtained at the lowest density of ∼4 μm^−2^ (sample for Au 16.0 nm). Therefore, the hydrophobicity of ZnO NRAs is attributed to the low surface free energy of the (001) plane of the ZnO nanorods, the special nano surface structure, and the roughness.^90,91^ These findings revealed that the water contact angle can be adjusted by changing NR array density.^91,92^ From these results it can be suggested that low contact angle NR arrays can be used for photocatalytic activity and in solar cells, and high contact angle can be used in field emission and waterproof devices.

## 4. Conclusions

In summary, ZnO NRA density can be controlled in a wide range of substrates using Ti and Au buffer layers by a facile, low cost, simple hydrothermal growth process. The NRAs grown on FTO and ITO substrates have more uniform distribution, and comparatively smaller diameter as well as higher NRA density than that grown on bare AZO and AZO/ZnO substrates. The ZnO NRA surface is more hydrophilic for Ti buffer layers than Au films due to the superhydrophobic properties of Au. The contact angle changes with the density variation presumably owing to the increase of roughness and groove captive air volume with decreasing NRA density. The static contact angle can be tuned from ∼23° to 135° and from ∼97° to 142° for Ti and Au buffer layers, respectively. The maximum CA obtained is ∼142° for Au buffer layer thickness (16.0 nm) corresponding to the lowest density of ZnO NRA density. A slight variation is observed of the FWHM and integrated intensity in XRD, Raman and PL spectra attributed to NRA density and/or size variation. These demonstrate that the control of ZnO NRA density using this technique is suitable for future device applications due to having no special requirements, working on transparent conducting substrates, and its scalability, low-cost and time-efficiency.

## Author contributions

M. Kamruzzaman and J. A. Zapien designed the study, interpreted the data, and wrote the manuscript. C. Y. Luan is a collaborator who helped to interpret the data, and wrote the manuscript.

## Conflicts of interest

The authors declare that they have no conflicts of interest.

## Supplementary Material

NA-005-D3NA00299C-s001
