# Supplementary material for: Effect of Ti and Au buffer layers on controlling the density and wettability of well-aligned ZnO nanorod arrays grown on different substrates
Source: Nanoscale Adv. 2023 Jun 28;5(14):3717–28. doi: 10.1039/d3na00299c (PMC10334411; doi:10.1039/d3na00299c)
Supplement: NA-005-D3NA00299C-s001 [file NA-005-D3NA00299C-s001.pdf]

Electronic Supplementary Material (ESI) for Nanoscale Advances.
This journal is © The Royal Society of Chemistry 2023

Supplement for

Effect of Ti and Au buffer layers on controlling the density and wettability of well-aligned ZnO nanorod arrays grown on different substrates

by

M. Kamruzzaman[1*] and J. A. Zapien[2*]

[1*]Department of Physics Begum Rokeya University, Rangpur, Rangpur-5400, Bangladesh
[2*]Department of Materials Science and Engineering, Center Of Super-Diamond and Advanced Films (COSDAF), City University of Hong Kong, Hong Kong SAR, P. R. China.
Email: kzaman.phy11@gmail.com (Tel: +880-1771034439), apjazs@cityu.edu.hk
(Tel: +852-3442-7823).

Figures S1 and S2 presented the side views FE-SEM of ZnO nanorod arrays grown on modified bare AZO, AZO/ZnO, FTO/ZnO and ITO/ZnO substrates using Ti and Au buffer layers. Inset Figures S1 and S2 also include the corresponding digital images used for the contact angle measurements. It is seen that the morphology, arrays density and hence the wettability changed as function of buffer layers thickness. The maximum contact angle is obtained for the lowest NR arrays density.

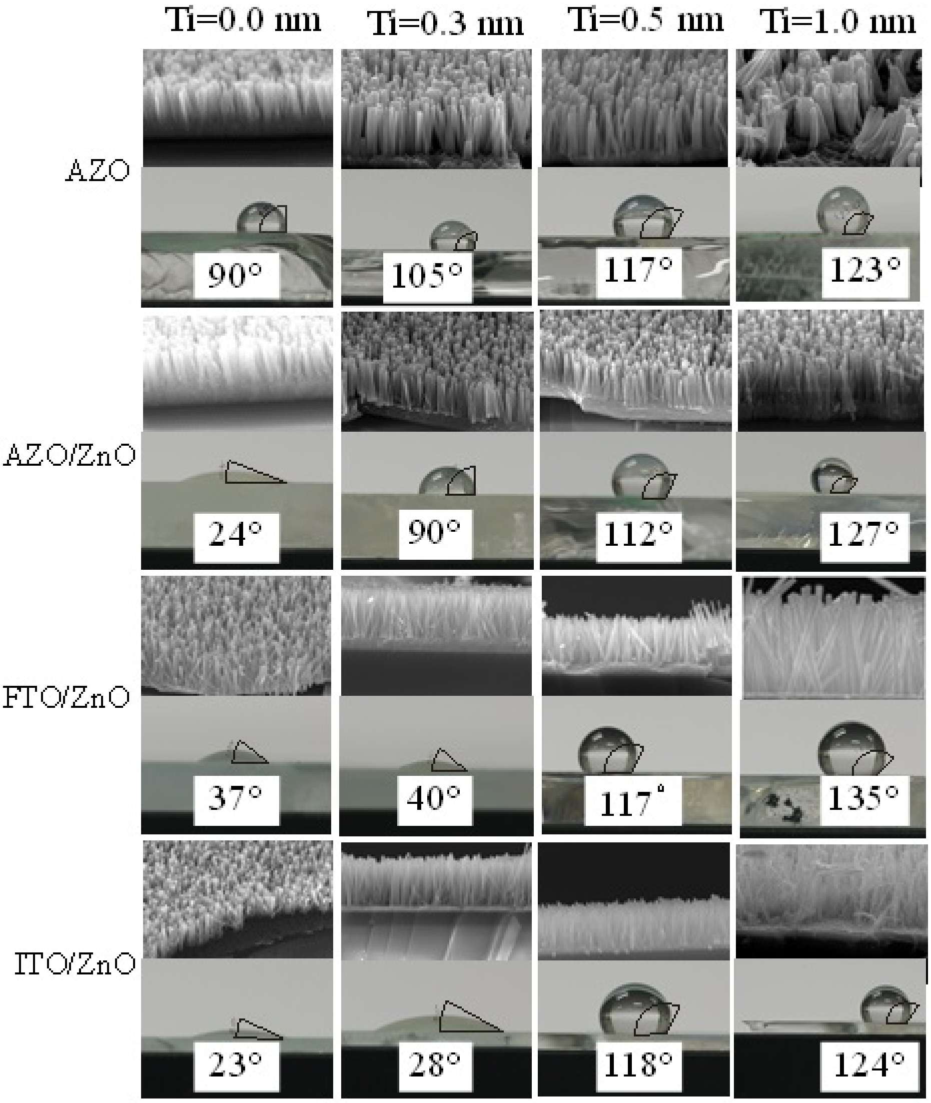

Figure S1 Side-view FE-SEM images of ZnO NRAs on bare AZO (1st row), AZO/ZnO (2nd row), FTO/ZnO (3rd row) and ITO/ZnO (4th row) for the Ti buffer layer of different thickness, presented in each column, for the control data (no-Ti buffer, 0.0 nm Ti, in the left-most column) and increasing Ti buffer layer thickness of columns 2 to 4 corresponding to the Ti thickness of 0.3, 0.5, and 1.0 nm, respectively. The corresponding below rows represent the digital image used in the static contact angle measurements.

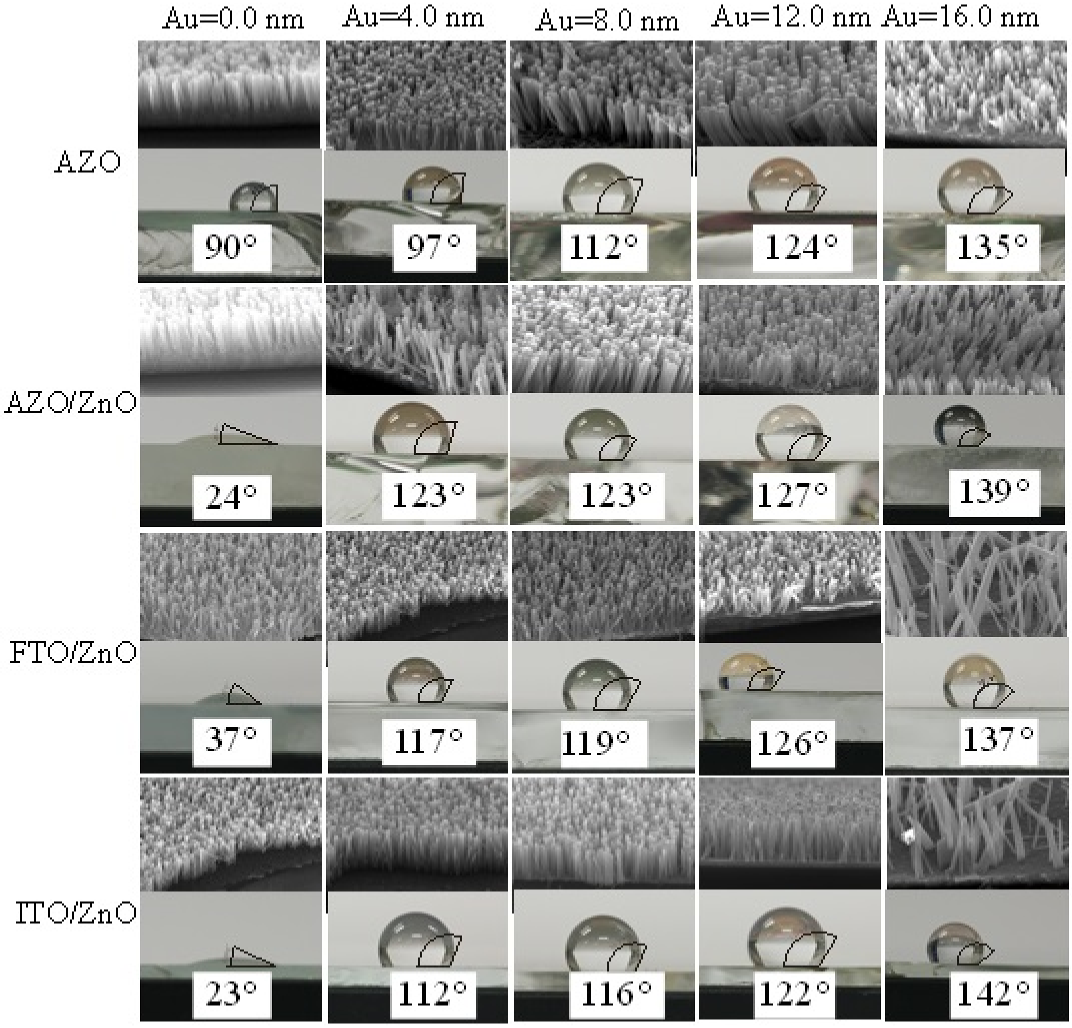

Figure S2 Side-view FE-SEM images of ZnO NRAs on bare AZO (1st row), AZO/ZnO (2nd row), FTO/ZnO (3rd row), and ITO/ZnO (4th row) for the Au buffer layer of different thickness, presented in each column for the control data (no -Au buffer, 0.0 nm Au, in the left-most column) and increasing Au buffer layer thickness of columns 2 to 5 corresponding to the Au thickness of 4.0, 8.0, 12.0, and 16.0 nm, respectively. The corresponding below rows represent the digital image used in the static contact angle measurements.

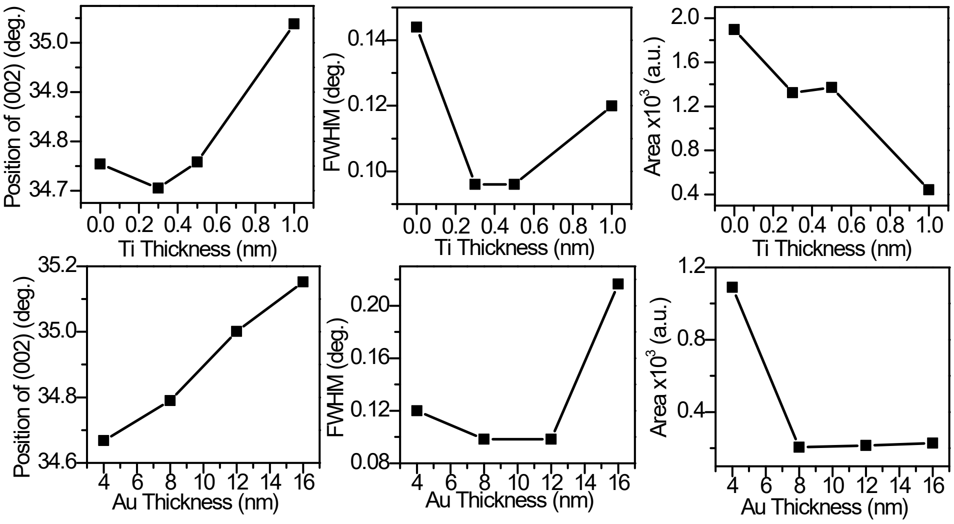

Figure S3 Variation of the (002) peak position, FWHM and integrated intensity with the increase of Ti (top row) and Au (below row) thickness for FTO/ZnO substrate, respectively.

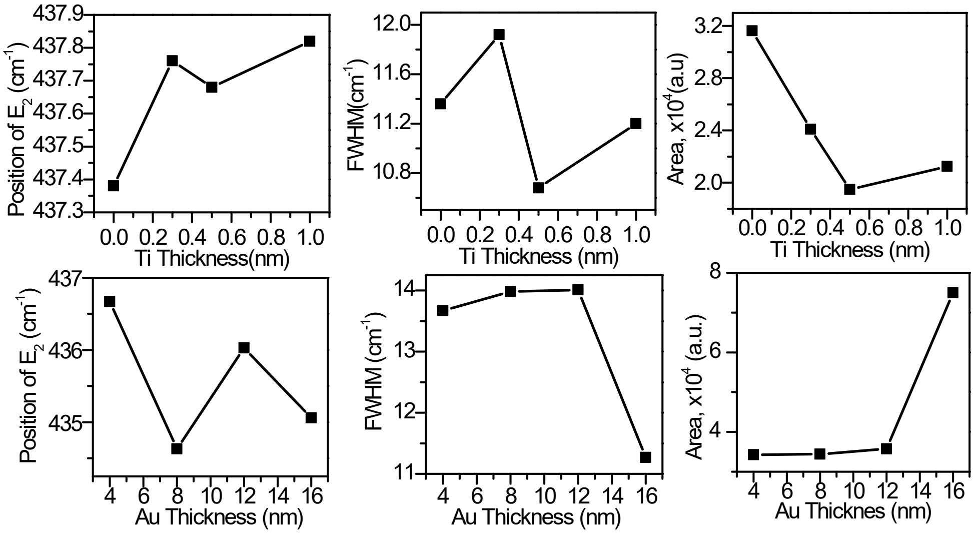

Figure S4 Variation of the E2 (high) band position, FWHM and integrated intensity with the increase of Ti (top row) and Au (below row) thickness for FTO/ZnO substrate, respectively.

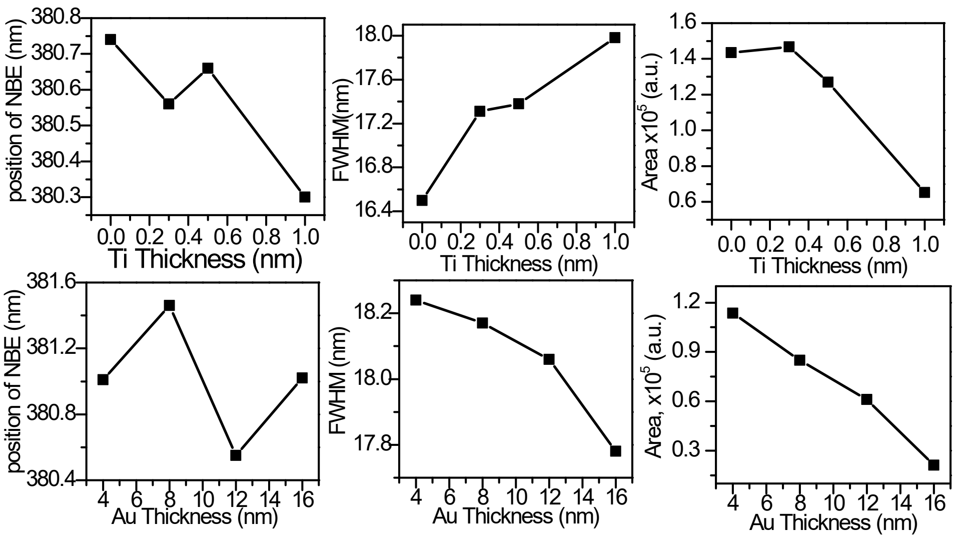

Figure 5S Variation of the NBE peak position, FWHM and integrated intensity with the increase of Ti (top row) and Au (below row) thickness for FTO/ZnO substrate, respectively.

Table S1. Variation of different parameters of ZnO NR arrays density control with Ti film thickness.

Substrate	Seed on substrate	Ti thickness (nm) (control data)	Average diameter (nm)	NRs Length (µm)	NRs density (μm^{-2})	Contact angle (degree)
AZO	No seed	0.0	153	0.88	28	90.00
		0.3	128	1.53	24	105.57
		0.5	136	1.48	17	117.26
		1.0	155	1.42	9	123.23
AZO	ZnO	0.0	96	1.10	43	24.49
		0.3	99	1.13	38	90.00
		0.5	108	0.96	35	113.42
		1.0	121	0.78	28	127.82
FTO	ZnO	0.0	62	1.20	100	37.09
		0.3	76	1.45	48	40.91
		0.5	79	1.52	48	117.53
		1.0	135	3.67	15	135.0
ITO	ZnO	0.0	70	1.27	75	23.86
		0.3	90	1.40	50	28.07
		0.5	97	1.53	46	118.18
		1.0	103	1.66	38	124.40

Table S2: Variation of different parameters of ZnO NR arrays density control with Au film thickness.

Substrate	Seed on substrate	Au thickness (nm) (control data)	Average diameter (nm)	NRs Length (µm)	NRs density (μm^{-2})	Contact angle (degree)
AZO	No seed	0	153	0.88	28	90.00
		4	134	0.95	25	97.32
		8	155	1.43	18	112.28
		12	194	1.57	13	124.29
		16	137	1.17	10	135.00
AZO	ZnO	0.0	96	1.10	43	24.49
		4	103	1.38	36	123.69
		8	113	1.57	29	123.14
		12	107	1.40	25	127.29
		16	104	1.30	18	139.01
FTO	ZnO	0.0	62	1.2	100	37.09
		4	83	0.77	54	117.29
		8	89	1.2	44	119.41
		12	104	1.18	37	126.00
		16	110	2.00	12	137.89
ITO	ZnO	0.0	70	1.27	75	23.86
		4	98	0.88	48	112.62
		8	102	1.06	42	116.71
		12	111	1.20	36	122.55
		16	140	1.29	4	142.12
